# Dasatinib Accelerates Valproic Acid-Induced Acute Myeloid Leukemia Cell Death by Regulation of Differentiation Capacity

**DOI:** 10.1371/journal.pone.0098859

**Published:** 2014-06-11

**Authors:** Sook-Kyoung Heo, Eui-Kyu Noh, Dong-Joon Yoon, Jae-Cheol Jo, Jae-Hoo Park, Hawk Kim

**Affiliations:** 1 Biomedical Research Center, Ulsan University Hospital, University of Ulsan College of Medicine, Ulsan, Republic of Korea; 2 Division of Hematology and Hematological Malignancies, Department of Hematology and Oncology, Ulsan University Hospital, University of Ulsan College of Medicine, Ulsan, Republic of Korea; Westmead Millennium Institute, University of Sydney, Australia

## Abstract

Dasatinib is a compound developed for chronic myeloid leukemia as a multi-targeted kinase inhibitor against wild-type BCR-ABL and SRC family kinases. Valproic acid (VPA) is an anti-epileptic drug that also acts as a class I histone deacetylase inhibitor. The aim of this research was to determine the anti-leukemic effects of dasatinib and VPA in combination and to identify their mechanism of action in acute myeloid leukemia (AML) cells. Dasatinib was found to exert potent synergistic inhibitory effects on VPA-treated AML cells in association with G_1_ phase cell cycle arrest and apoptosis induction involving the cleavage of poly (ADP-ribose) polymerase and caspase-3, -7 and -9. Dasatinib/VPA-induced cell death thus occurred via caspase-dependent apoptosis. Moreover, MEK/ERK and p38 MAPK inhibitors efficiently inhibited dasatinib/VPA-induced apoptosis. The combined effect of dasatinib and VPA on the differentiation capacity of AML cells was more powerful than the effect of each drug alone, being sufficiently strong to promote AML cell death through G_1_ cell cycle arrest and caspase-dependent apoptosis. MEK/ERK and p38 MAPK were found to control dasatinib/VPA-induced apoptosis as upstream regulators, and co-treatment with dasatinib and VPA to contribute to AML cell death through the regulation of differentiation capacity. Taken together, these results indicate that combined dasatinib and VPA treatment has a potential role in anti-leukemic therapy.

## Introduction

Acute myeloid leukemia (AML) remains one of the most difficult hematologic malignancies to treat [1]. Efforts to improve standard cytotoxic chemotherapy, the current approach to AML treatment, have been unsuccessful, thus necessitating the development of new chemotherapeutic agents that can remove or diminish leukemic blasts in AML effectively.

Dasatinib (BMS-354825) is an FDA-approved small molecular compound that was developed primarily to treat chronic myeloid leukemia (CML) as a multi-targeted tyrosine kinase inhibitor against wild-type BCR-ABL and SRC family kinases [2]. To date, the compound has demonstrated promising anti-leukemic activity in both patients with imatinib-resistant or -intolerant CML and those with newly diagnosed CML [3]–[5]. The off-target effects of tyrosine kinase inhibitors, including dasatinib, on AML differentiation have attracted considerable research interest in the past few years. For example, imatinib, the first BCR/ABL inhibitor, was discovered to exert an effect on the potentiation of all-trans-retinoic acid (ATRA)-induced AML differentiation [6], and the epidermal growth factor receptor inhibitor gefitinib was later confirmed to enhance the ATRA-induced differentiation of AML cells [7], [8]. Dasatinib demonstrated similar effects on such differentiation in a separate study [2].

Valproic acid (VPA) is a well-known anti-epileptic drug that is also a class I histone deacetylase inhibitor [9]. Interest in the use of such inhibitors as anti-cancer agents was recently sparked by research showing them to strongly induce cell cycle arrest, differentiation and malignant cell apoptosis [10]. There were also earlier reports of VPA inducing cell cycle arrest and apoptosis in hepatoma [11], prostate carcinoma [12] and thyroid cancer cells [13]. Studies have also revealed the anti-leukemic activity of VPA in human Philadelphia chromosome-positive acute lymphatic and CML cells [14] and in AML cells expressing P-glycoprotein and multidrug resistance-associated protein 1 [15].

However, little is known about the anti-leukemic effects of dasatinib or whether its use in combination with VPA would have a synergistic treatment effect. The purpose of the research reported herein was thus to determine the anti-leukemic effects of both dasatinib and VPA and to identify their mechanism of action in acute myeloid leukemia (AML) cells. We hypothesized that dasatinib and VPA in combination would exert synergistic effects on the apoptotic activity and G_1_ phase cell cycle arrest of AML cells.

## Materials and Methods

### Reagents

All of the reagents, including VPA, were obtained from Sigma-Aldrich (St. Louis, MO) unless otherwise indicated. The CellTiter 96 AQueous One Solution Cell Proliferation Assay (MTS) was purchased from Promega (Madison, WI), and RPMI 1640 medium and fetal bovine serum (FBS) from GibcoBRL (Grand Island, NY). Annexin V-FITC Apoptosis Detection Kit I, PI/RNase staining buffer, anti-human CD11b-PE, anti-human CD14-PE and mouse IgG_1_-PE were purchased from BD Biosciences (San Diego, CA). DRAQ5 was purchased from Abcam (Cambridge, MA). The Apoptosis Antibody Sampler Kit, anti-p27^kip1^, CDK4, CDK6 and cyclin D1 were purchased from Cell Signaling Technology (Beverly, MA). All of the inhibitors, including the mitogen-activated protein kinase (MAPK) inhibitors (U0126, PD98059, SB203580 and SP600125), caspase-3 inhibitor (Z-DEVD-FMK) and caspase-9 inhibitor (LEHD-CHO), were obtained from Merck Millipore (Billerica, MA). The ApoTarget Caspase-3 Protease Assay Kit for caspase-3 activity and CasGLOW Fluorescein Active Caspase-9 Staining Kit were purchased from Invitrogen (Camarillo, CA) and eBioscience (Atlanta, GA), respectively, and the Immun-star WesternC Kit was purchased from Bio-Rad (Hercules, CA). Finally, the Western antibodies, anti-p21^cip1^, CDK2, cyclin E, β-actin and anti-rabbit IgG-HRP were purchased from Santa Cruz Biotechnology (Santa Cruz, CA).

### Cells and Cell Culture

Human AML HL60, Kasumi-1 and NB4 cells were obtained from the American Type Culture Collection (ATCC, Manassas, VA). The HL60 and NB4 cells were grown as suspension cultures in 100-mm culture dishes in RPMI 1640 medium supplemented with 10% heat-inactivated FBS and 1% penicillin-streptomycin in a 5% CO_2_ humidified atmosphere at 37°C. The Kasumi-1 cells were also grown as suspension cultures in RPMI 1640 medium, but were supplemented with 20% heat-inactivated FBS, 4.5 g/L glucose, 2 mM L-glutamine and 1% penicillin-streptomycin in the same condition. Human hepatoma cell lines Hep G2 and Hep 3B and breast cancer cell line MCF-7 were purchased from the ATCC, and were grown as adherent cultures in 100-mm culture dishes in RPMI 1640 medium and Eagle’s Minimum Essential Medium supplemented with 10% heat-inactivated FBS and 1% penicillin-streptomycin in a 5% CO_2_ humidified atmosphere at 37°C.

### Patient Samples

Two patients recently diagnosed with AML (other diseases not specified) at Ulsan University Hospital, Ulsan, South Korea, participated in this study: patient AML-1, a 55-year-old woman, and patient AML-2, a 71-year-old woman. Blood and bone marrow samples were collected from both prior to their first round of chemotherapy.

### Ethics Statement

Both subjects provided informed written consent before the study’s commencement. The study protocol and patient consent form and information were approved by the Ulsan University Hospital Ethics Committee and Institutional Review Board (UUH-IRB-11-18).

### Isolation of Patient Cells

The peripheral blood and bone marrow samples obtained from the two subjects were drawn into heparinized tubes, and separated via density gradient centrifugation at 400×*g* using Lymphoprep (Axis-Shield, Oslo, Norway). Peripheral blood mononuclear cells (PBMC) and bone marrow cells (BMC) were isolated and washed with RPMI 1640 medium, and then cultured in 24-well culture plates in the same medium with 10% FBS and 1% penicillin-streptomycin in a 5% CO_2_ humidified atmosphere at 37°C. The cells were then subjected to a number of experiments, as described in the following.

### Cell Viability Assay

Cell proliferation and cytotoxicity were assessed with the CellTiter 96 AQueous One Solution Cell Proliferation Assay. All cells were seeded in 96-well plates at a density of 2×10^4^ cells/ml, with 100 µl of medium per well, and then incubated with 0.5 mM of VPA and 5 µM of dasatinib for 72 h at 37°C. In some of the experiments, the cells were cultured with various concentrations of VPA (0, 0.5, 1, 1.5 and 2 mM) and dasatinib (0, 1, 3, 5, 10 and 15 µM) for 72 h at 37°C. The CellTiter 96 solution (20 µl) was added directly to each well, and the plate was incubated for 4 h in a humidified 5% CO_2_ atmosphere at 37°C. Absorbance was measured with a PowerWave XS2 Microplate Spectrophotometer (BioTek, Winooski, VT) at 490 nm, and the results were expressed as percentage changes from the base conditions using four to five culture wells for each experimental condition.

### Cell Cycle Analysis

The HL60 cells (5×10^5^ cells/ml) were seeded in 24-well plates, and treated with 0.5 mM of VPA and/or 5 µM of dasatinib for 24, 48 and 72 h at 37°C. They were washed twice with phosphate buffered saline (PBS), and fixed with 70% ethanol for 4 h at −4°C, and then washed again with PBS and incubated with 0.5 ml of PI/RNase stain buffer and incubated for 15 min at room temperature. The samples were then analyzed with a FACSCalibur flow cytometer and CellQuest Pro software (BD Biosciences).

### Western Blotting of Cell Cycle- and Caspase-related Proteins

Samples of p21^Cip1^, p27^Kip1^, CDK2, CDK4, CDK6, cyclin D1 and cyclin E were cultured for 72 h, and samples of procaspase-3, -7, -9 and cleaved caspase-3, -7 and -9 for 96 h. Total cell extracts were prepared using RIPA buffer. Equal amounts of cell extract (40–80 µg) were resolved on sodium dodecyl sulfate polyacrylamide gel electrophoresis, and electro-transferred to nitrocellulose membranes for 1.5 h. The membranes were blocked with 4% nonfat dried milk in PBS-T (0.05% Tween-20) buffer for 1 h and blotted with their respective primary antibodies for 2 h. They were subsequently washed three times with PBS-T for 10 min each, and then incubated with their respective horseradish peroxidase (HRP)-conjugated secondary antibodies for 1 h. Finally, the membranes were developed using the Immun-star WesternC kit.

### Annexin V and Propidium Iodide Staining

All of the cell types, including the HL60 cells, PBMC and BMC (5×10^5^ cells/ml), were cultured with 0.5 mM of VPA and/or 5 µM of dasatinib for 72 h at 37°C. They were then washed twice with FACS buffer (PBS containing 0.3% BSA and 0.1% NaN_3_), incubated with annexin V-FITC and propidium iodide (PI) from Apoptosis Detection Kit I, and finally analyzed using the FACSCalibur flow cytometer and CellQuest Pro software according to the manufacturer’s protocol. In the experiments in which we used several inhibitors to prevent caspase or MAPK activation, the cells were pre-incubated with the caspase and MAPK inhibitors for 1 h at 37°C before the addition of dasatinib/VPA.

### DRAQ5 Nuclear Staining

Cells were incubated with 0.5 mM of VPA and/or 5 µM of dasatinib for 72 h at 37°C, and then harvested and washed twice with PBS buffer. For DNA content analysis of the nuclei, the cells were stained with 5 µM of DRAQ5 and incubated for 30 min at room temperature. The manufacturer describes DRAQ5 as a cell-permeable far-red fluorescent DNA dye that can be used in live and fixed cells. In our experiments, the stained cells were prepared using FlowSight and analyzed with IDEAS software (Merck Millipore).

### Intracellular Staining of Cleaved Poly (ADP-ribose) Polymerase (PARP) and Cleaved Caspase-3

Cells were incubated with 0.5 mM of VPA and/or 5 µM of dasatinib for 72 h at 37°C, then harvested and washed twice with FACS buffer. Next, they were fixed with 4% paraformaldehyde in PBS, after which they were added to a solution of 0.1% Triton X-100 in PBS for permeabilization, as described in our previous report [16]. The cells were stained with anti-cleaved PARP, anti-cleaved caspase-3 mAb or isotype control mAb at 4°C for 30 min. The samples were then analyzed with the FACSCalibur flow cytometer and CellQuest Pro software. We also stained the cell nuclei with DRAQ5 (5 µM) and then analyzed the stained cells with FlowSight and IDEAS software.

### Measurement of Caspase-3 and -9 Activity

Cells were incubated with 0.5 mM of VPA and/or 5 µM of dasatinib for 72 h at 37°C, then harvested and washed twice with PBS buffer. Caspase-3 activity was measured using the ApoTarget assay kit, and absorbance with the PowerWave spectrophotometer at 400 nm. Caspase-9 activity was measured using the CasGLOW staining kit. Finally, the cells were analyzed with the FACSCalibur flow cytometer and CellQuest Pro software, and the results were expressed as the percentage of positive cells.

### Flow Cytometric Analysis

For flow cytometric analysis, cells were collected and treated in the same conditions as those described in the foregoing experiments. They were washed twice with FACS buffer and incubated with appropriate fluorochrome-labeled mAbs, such as anti-human CD11b-PE and CD14-PE or isotype control mAb, for 30 min at 4°C. The samples were then washed three times with FACS buffer and analyzed using the FACSCalibur flow cytometer and CellQuest Pro software, with the results again expressed as the percentage of positive cells.

### Statistical Analysis

All data presented herein represent the means ± standard error of mean (SEM) of at least three independent experiments. All values were evaluated via one-way analysis of variance (ANOVA) followed by Tukey’s range test using GraphPad Prism 6.0 software (San Diego, CA). Differences were considered significant at p<0.05.

## Results

### Dasatinib and VPA Regulate Differentiation Capacity Differently

**Figure 1:**
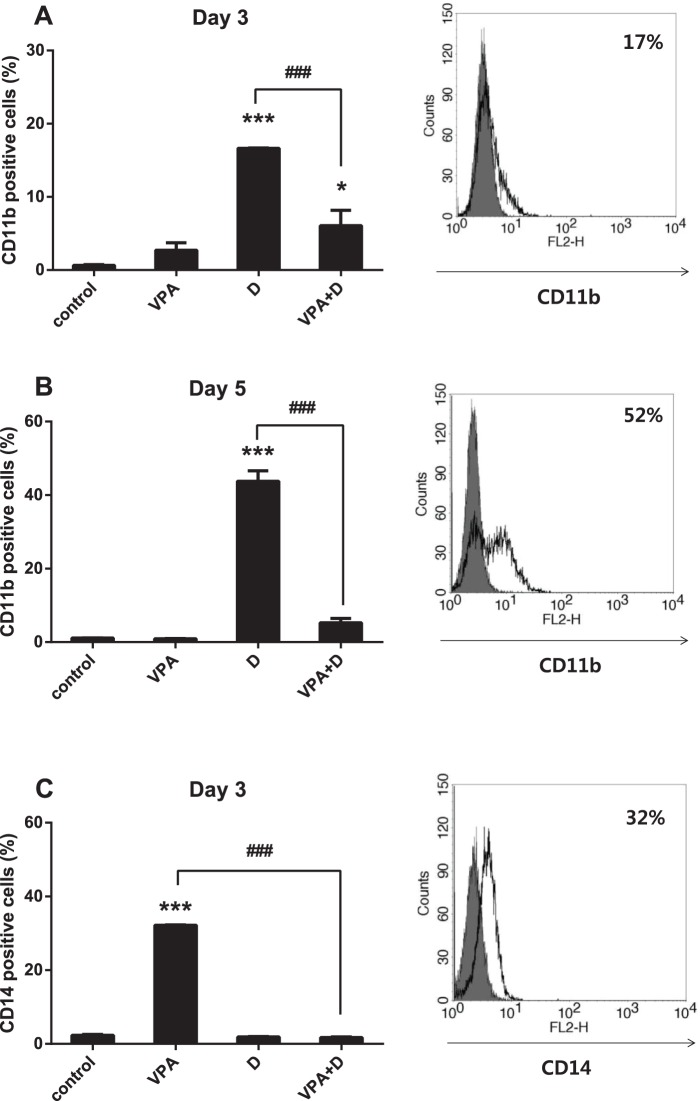
Effects of dasatinib and VPA on CD11b and CD14 expression in HL60 cells. Cells were incubated with 5 µM of dasatinib and 0.5 mM if VPA for 3 and 5 days. The cells were then harvested and immune stained with anti-human CD11b and CD14 mAb. The expression of CD11b and CD14 was then measured by flow cytometry. The filled histogram represents the isotype control, and the open histogram represents CD11b-positive cells treated with 5 µM if dasatinib alone at Day 3 (A) and Day 5 (B). The open histogram represents CD14-positive cells treated with 0.5 mM of VPA alone at Day 3 (C). These data represent the means ± SEM. Significantly different from the DMSO-treated control (*) or combination of VPA and dasatinib (#); ***, ###: *P*<0.001. VPA, valproic acid; D, dasatinib.

We examined the effects of dasatinib and VPA on differentiation markers and the cell surface expression of CD11b and CD14. The cells were treated with various concentrations of VPA and dasatinib for 72 h, with the differentiation markers then tested via flow cytometry. CD11b expression increased after exposure to dasatinib alone at days 3 and 5. However, combined dasatinib and VPA treatment led to a marked decrease on CD11b expression in HL60 cells, and the change occurred in a time-dependent manner (Figs. 1A and B). CD14 expression, in contrast, increased after exposure to VPA alone at day 3, whereas its combination with dasatinib resulted in a marked decrease in expression (down to the basal level) in HL60 cells (Fig. 1C).

### VPA-dasatinib Combination Induces AML Cell Death

**Figure 2:**
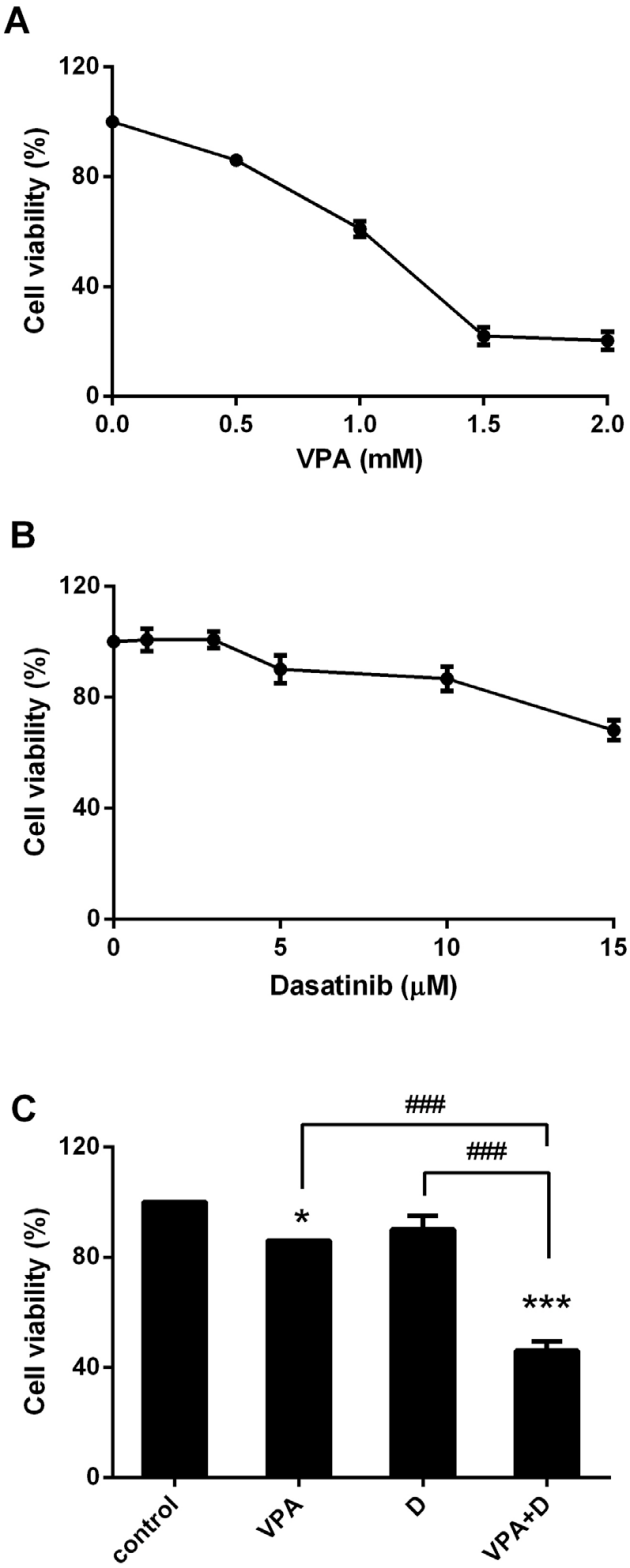
Combination of dasatinib and VPA inhibits HL60 cell proliferation. Cells were stimulated with various concentrations of 0, 0.5, 1, 1.5 and 2 µM dasatinib for 72 hr. The cytotoxicity was then evaluated by an MTS assay. (A) Dose-dependent responses of VPA on cell viability. (B) Dose-dependent responses of dasatinib on cell viability. (C) Treatment of VPA and/or dasatinib at 72 hr. Representative data are shown for at least three independent experiments. These data represent the means ± SEM. Significantly different from the control (*) or combination of VPA and dasatinib (#); *: *P*<0.05; ***, ###: *P*<0.001.

As noted previously, in some of the experiments the cells were treated with various concentrations of VPA (0, 0.5, 1, 1.5 and 2 mM) and dasatinib (0, 1, 3, 5, 10 and 15 µM). VPA and dasatinib significantly inhibited the viability of the HL60 cells in a dose-dependent manner (Figs. 2A and B). Interestingly, however, although 0.5 mM of VPA and 5 µM of dasatinib alone had little effect on the viability of these cells (over 85% and 90% cell viability, respectively), in combination these concentrations of VPA and dasatinib produced a significant inhibitory effect (46%; see Fig. 2C). Accordingly, we used these concentrations for the remainder of the experiments.

Our next task was to determine whether the aforementioned effects are AML-specific. We thus tested the combined effects of VPA and dasatinib on two additional AML cell lines with a different genetic phenotype, namely, NB4 and Kasumi-1, and on several non-AML cell lines, including hepatoma (HepG2 and Hep3B) and breast cancer (MCF-7) lines. NB4 cells belong to French-America-British (FAB) classification M3, and thus express the PML-RARA protein. Both Kasumi-1 and HL60 cells belong to FAB classification M2, but are different genetic phenotypes, with only the former expressing the AML1-ETO protein. We conducted an experiment to detect the effects of the VPA and dasatinib combination on the viability of all of these cell lines. As shown in Table 1, the combination exerted prominent effects on the viability of the AML cell lines, including Kasumi-1, NB4 and HL60, whereas both hepatoma cell lines died following treatment with dasatinib alone. Conversely, the MCF-7 cells proliferated following treatment with VPA, dasatinib or a combination of the two. These results indicate that the synergistic effects of the VPA and dasatinib combination do indeed appear to be AML-specific.

**Table 1:** Effects of VPA and dasatinib on the cell viability.

Cell lines	Control	VPA	D	VPA + D
Kasumi-1	100±0.0	60±1.5***^, ###^	37±3.2***^, ###^	16±3.6***
NB4	100±0.0	46±2.5***^, ###^	40±2.2***^, ###^	24±1.2***
HL60	100±0.0	86±0.5*^, ###^	90±5.0^###^	46±3.4***
HepG2	100±0.0	95±2.4	90±2.5*	97±2.0
Hep3B	100±0.0	108±3.0^###^	53.7±2.5***	49±2.9***
MCF-7	100±0.0	132±13	150±2.8***	149±4.8***

These data represent the means ± SEM. Significantly different from control (*) or combination of VPA and D (#); ***, ###: *P*<0.001. *: *P*<0.05. VPA, Valproic acid; D, dasatinib.

### Dasatinib Accelerates G_1_ Phase Cell Cycle Arrest in VPA-treated HL60 Cells

**Figure 3:**
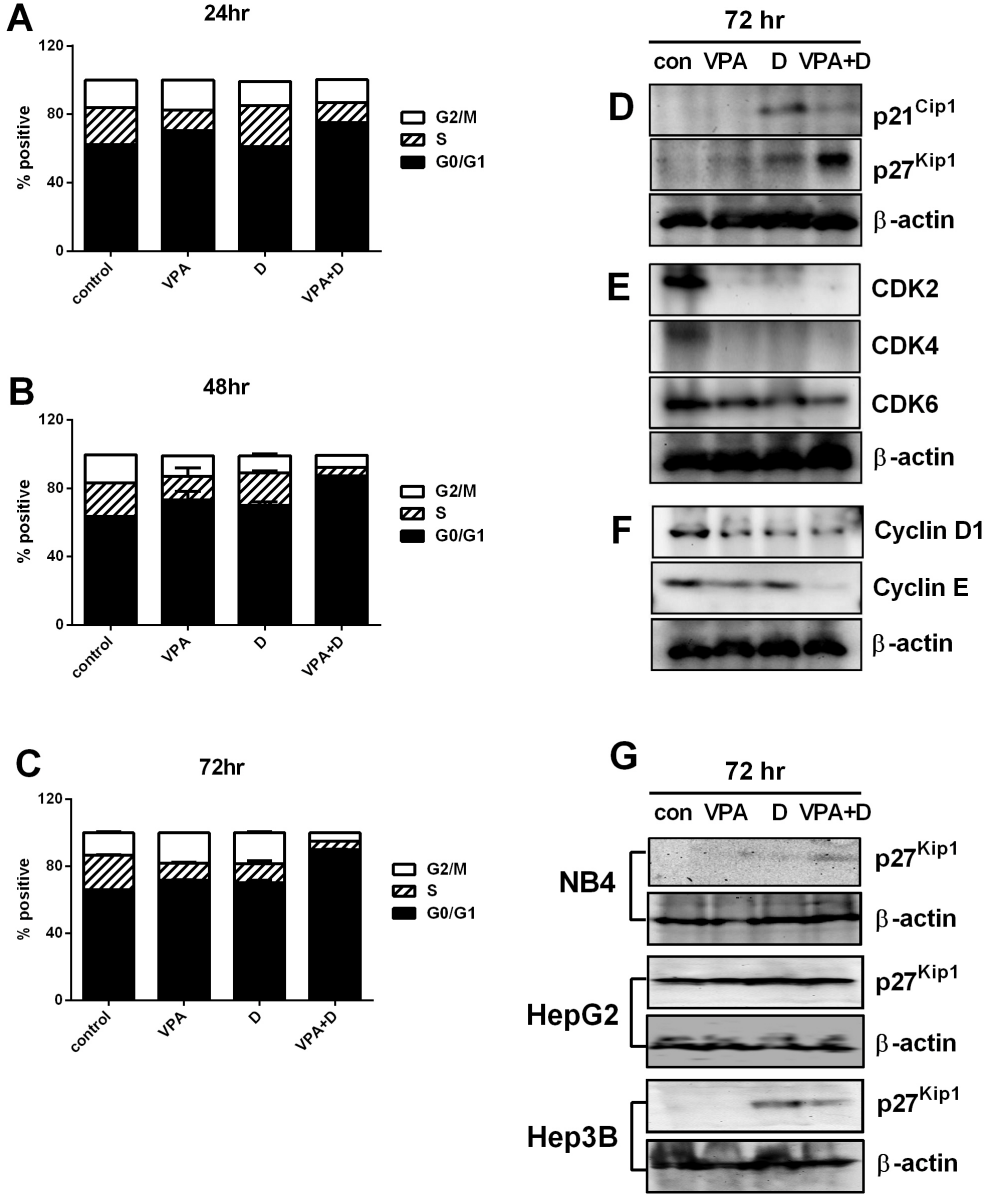
Synergistic effects of dasatinib and VPA on G_1_ phase cell cycle arrest. Cells were incubated with 0.5 µM of dasatinib for 72 hr. The cells were harvested at 24 hr (A), 48 hr (B) and 72 hr (C) and then stained with PI/RNase staining buffer and analyzed by flow cytometry. The expression of G_1_ phase cell cycle regulatory proteins was then measured by Western blot analysis. The membrane was stripped and reprobed with anti-β-actin mAb to confirm equal loading. (D) The expression of p21^Cip1^ and p27^Kip1^. (E) The expression of CDK2, 4 and 6. (F) The expression of cyclin D1 and E. (G) The expression of p27^Kip1^ on NB4, HepG2, and Hep3B. Representative blots are shown from three independent experiments with similar pattern results.

As shown in Figure 2, we observed the VPA-dasatinib combination to have a strong growth-inhibitory effect in the HL60 cells. Accordingly, we investigated the possible mechanism of this anti-proliferative activity, and also tested the effects of VPA (0.5 mM) and dasatinib (5 µM) on cell cycle progression in these cells. Figure 3 shows that the dasatinib-VPA combination resulted in a significantly higher percentage of G_0_/G_1_ phase cells in a time-dependent manner. In comparison with the control group, the percentage increase in cells in the G_0_/G_1_ phase was 13% at 24 h, 23% at 48 h and 24% at 72 h. The percentages of G_1_ cells arrested were 63.5% (control), 71% (VPA), 70% (dasatinib) and 87% (combination) at 48 h (Fig. 3B) and 66% (control), 71.5% (VPA), 70.5% (dasatinib) and 90% (combination) at 72 h (control versus combination at 72 h, *p*<0.001; Fig. 3C). Treatment with each drug alone also increased the number of arrested cells, but not to a statistically significant degree (less than 5% compared with the control group). The response to the combination treatment in terms of cell cycle progression was almost saturated at 48 h, and the signal patterns were very similar to those at 72 h. The results again revealed the level of G_0_/G_1_ arrest to be higher than 90% in the HL60 cells at 72 h (Fig. 3A–C).

### VPA-dasatinib Combination Increases p21^Cip1^ and p27^Kip1^ Expression in HL60 Cells

Cyclin-dependent kinases (CDKs) are serine/threonine kinases whose catalytic activities are controlled by interactions with cyclins and CDK inhibitors (CKIs) [17]. CKIs also regulate cell progression in the G_1_ phase of the cell cycle. The induction of p21^Cip1^ and p27^Kip1^, two well-known CKIs, is associated with blocking of the G_1_ and S transition, which in turn results in G_0_/G_1_ phase arrest in the cell cycle [18]. Because the stimulation of HL60 cells with VPA and dasatinib induced G_0_/G_1_ arrest, as shown in Figure 3, we next analyzed the two drugs’ effects on the cell cycle regulatory proteins involved in the G_1_ phase of cell cycle progression, including CDKs, cyclins and CKIs. After stimulating the HL60 cells with 0.5 mM of VPA and/or 5 µM of dasatinib for 72 h, we determined the expression of p21^Cip1^ and p27^Kip1^ using Western blotting. Figure 3D shows the expression of the two following combination treatment to be 59- and 55-fold greater, respectively, than the control values, as we expected. However, the effect of dasatinib alone on p21^Cip1^ expression was 18% higher than that of the combination treatment, and VPA seemed to reduce the dasatinib-induced p21^Cip1^ levels (a 72-fold increase in p21^Cip1^ band density with dasatinib alone versus a 59-fold increase with the combination). These results suggest that combined VPA-dasatinib treatment increases the expression of inhibitory proteins p21^Cip1^ and p27^Kip1^ in HL60 cells, consequently keeping those cells in the G_1_ phase (Fig. 3D).

### VPA-dasatinib Combination Decreases Expression of G_1_ Phase Cell Cycle Regulatory Proteins, CDKs and Cyclins in HL60 Cells

Several studies have shown CDKs and cyclins to play important roles in the regulation of cell cycle progression [18], [19]. In this research, we confirmed the effect of combined VPA-dasatinib treatment on the expression of CDKs and cyclins, which are negatively regulated by p21^Cip1^ and p27^Kip1^ during G_1_ arrest in the cell cycle progression. We also assessed the effects of VPA and dasatinib on CDK2, CDK4 and CDK6 and cyclins D1 and E in the same conditions as those reported above. Figure 3E shows that the combination of the two led to a decrease in the expression of CDK2, CDK4 and CDK6, and the band density observed for CDK2 was 1/150-fold lower than that of the control. A similar marked reduction in cyclin D1 and E expression was observed at 72 h (Fig. 3F). The synergistic effects of VPA and dasatinib on the expression of G_1_ phase cell cycle regulatory proteins thus appear to be regulated by the CKI-CDK-cyclin cascade in HL60 cells (Figs. 3D–F).

We also observed the expression of p27^Kip1^ in the NB4, HepG2 and Hep3B cells. As shown in Figure 3G, VPA and dasatinib were found to exert synergistic effects on the AML and NB4 cells alone. The effects of the combination treatment appear to be dominant on AML cells.

### Dasatinib Induces Apoptosis in VPA-treated AML Cells

**Figure 4:**
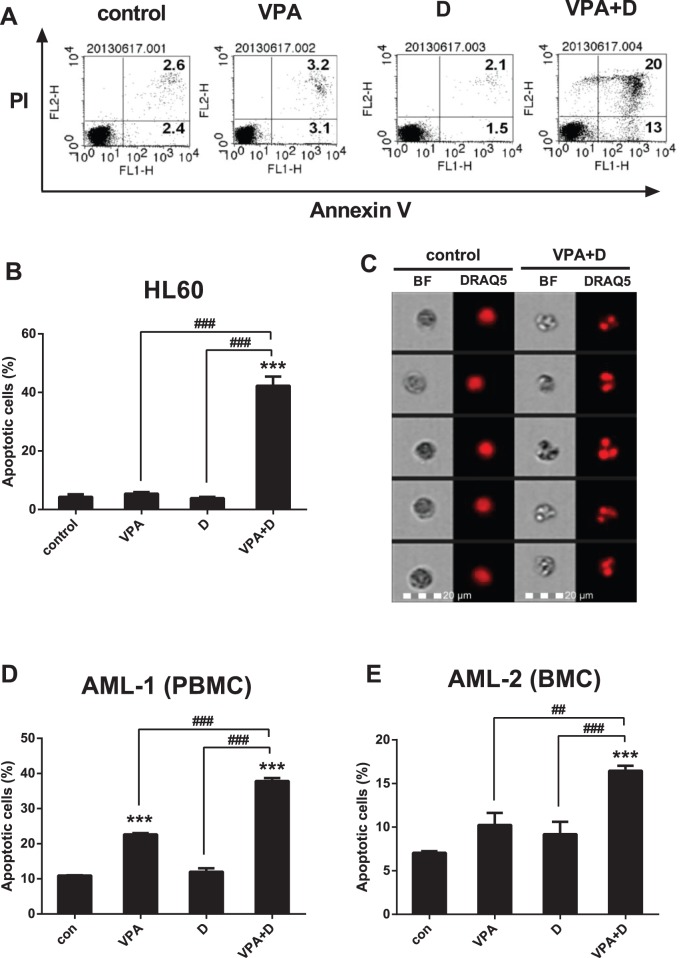
Dasatinib induces apoptosis in VPA-treated AML cells. The cells were also collected and treated under the same conditions described in Figure 3. Cells were stained with annexin V-FITC and/or propidium iodide (PI) followed by flow cytometry analysis. (A) Annexin V/PI staining of HL60 cells. (B) Data show the percentage of annexin V-positive cells (apoptotic cells) on (A). (C) DRAQ5 nuclear staining following combination treatment in HL60 cells. Data show the percentage of apoptotic cells of PBMC (D) and BMC (E) in the AML patients. These data represent the means ± SEM. Significantly different from the control (*) or combination of VPA and dasatinib (#); ##: *P*<0.01; ***, ###: *P*<0.001.

Apoptosis was measured by the annexin V binding of phosphatidylserine following treatment with 0.5 mM of VPA and/or 5 µM of dasatinib, with combined treatment found to induce apoptosis in the HL60 cells (Figs. 4A and B). As shown in Figure 4C, the nuclei of the combination group cells were divided into several fragments. We further investigated the effects of dasatinib and VPA on the PBMC and BMC obtained from the two AML patients. The PBMC from patient AML-1 contained 60% blast cells, and the BMC from patient AML-2 contained 82%. Results similar to those in Figure 4B were found in primary culture cells from the two patients (Figs. 4D and E). However, the sensitivities of PBMC and BMC following VPA treatment were slightly higher than those of the HL60 cells.

We monitored the combined effects of VPA and dasatinib on apoptotic cells in the same conditions as those listed in Table 1. Table 2 shows the effects of the VPA and dasatinib combination on apoptosis to have been most prominent in the Kasumi-1, NB4 and HL60 AML cells. These effects were not observed in the solid cancer cells, i.e., HepG2, Hep3B or MCF-7. These results again confirm the synergistic effects of the VPA and dasatinib combination on AML cells.

**Table 2:** Effects of VPA and dasatinib on the apoptotic cells.

Cell lines	Control	VPA	D	VPA + D
Kasumi-1	7.5±0.2	16.0±0.5***^, ###^	61.0±1.1***^, ###^	92.0±0.9***
NB4	6.5±0.9	24.8±4.1*^, #^	21.0±2.8**^, ###^	58.6±4.4***
HL60	4.3±0.9	5.4±0.5^###^	3.8±0.4^###^	42.2±3.1***
HepG2	10.0±0.4	13.7±1.2	20.2±3.9*	18.0±1.4
Hep3B	8.6±0.9	3.3±0.4***^, ###^	21.5±0.9***	18.5±1.0***
MCF-7	2.7±0.3	4.8±0.3*	2.8±0.2	3.1±0.2

These data represent the means ± SEM. Significantly different from control (*) or combination of VPA and D (#); ***, ###: *P*<0.001. **: *P*<0.01. *, #: *P*<0.05. VPA, Valproic acid; D, dasatinib.

### Synergic Effects of Dasatinib and VPA on PARP and Caspase-9, -3 and -7 Activations in HL60 Cells

**Figure 5:**
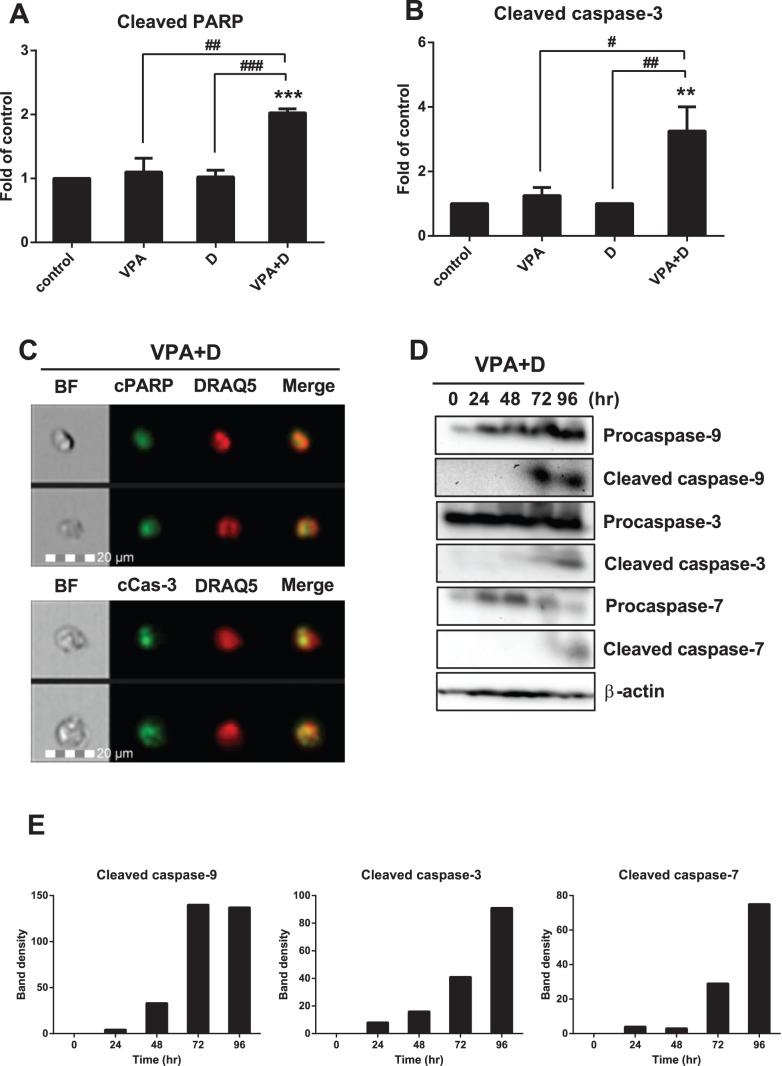
Dasatinib/VPA-induced apoptosis activates PARP and caspase-9, -3 and -7 in HL60 cells. Cells were collected and treated under the same conditions described in Figure 3. The cells were intracellular stained with anti-human cleaved PARP (cPARP), anti-human cleaved caspase-3 (cCas-3) and anti-rabbit IgG-FITC, followed by flow cytometry analysis. (A) The expression of intracellular cPARP. (B) The expression of intracellular cCas-3. (C) The intracellular expression of cPARP and cCas-3 in the combination group was monitored by FlowSight analysis. (D) The expression of capsase-9, -3 and -7 and procapsase-9, -3 and -7 was then measured by Western blot analysis. The membrane was stripped and reprobed with anti-β-actin mAb to confirm equal loading. (E) Data show the band density of (D). Representative blots are shown from three independent experiments with almost identical results. These data represent the means ± SEM. Significantly different from control (*) or combination of VPA and dasatinib (#); #: *P*<0.05; **, ##: *P*<0.01; ***, ###: *P*<0.001.

Caspase activation is a principal feature of apoptosis, with the key factors of this mechanism conserved throughout evolution [20]. Caspase-9 and -3 are known to play crucial roles in the terminal phase of apoptosis [16]. To determine the dasatinib-induced apoptosis pathway in VPA-activated HL60 cells, we examined the expression of intracellular cleaved PARP and cleaved caspase-3. As shown in Figures 5A and B, the expression of both was significantly induced by the combination of VPA and dasatinib. Intracellular cleaved PARP and cleaved caspase-3 expression was also monitored in the combination group with the FlowSight imaging system, with patterns similar to those in Figures 5A and B observed (Fig. 5C). The nuclei were then stained with DRAQ5 dye as a positive control, and we next confirmed the protein levels of both procaspase-9, -3 and -7 and cleaved caspase-9, -3 and -7. All of the cleaved caspases were activated via VPA and dasatinib stimulation in a time-dependent manner (Figs. 5D and E). The results indicate that activation of a series of caspases (caspase-9, -3, -7) and PARP is a necessary condition for dasatinib/VPA-induced apoptosis in HL60 cells (Fig. 5).

### Caspase-9 and -3 are Essential to Dasatinib/VPA-induced Apoptosis Pathway in HL60 Cells

**Figure 6:**
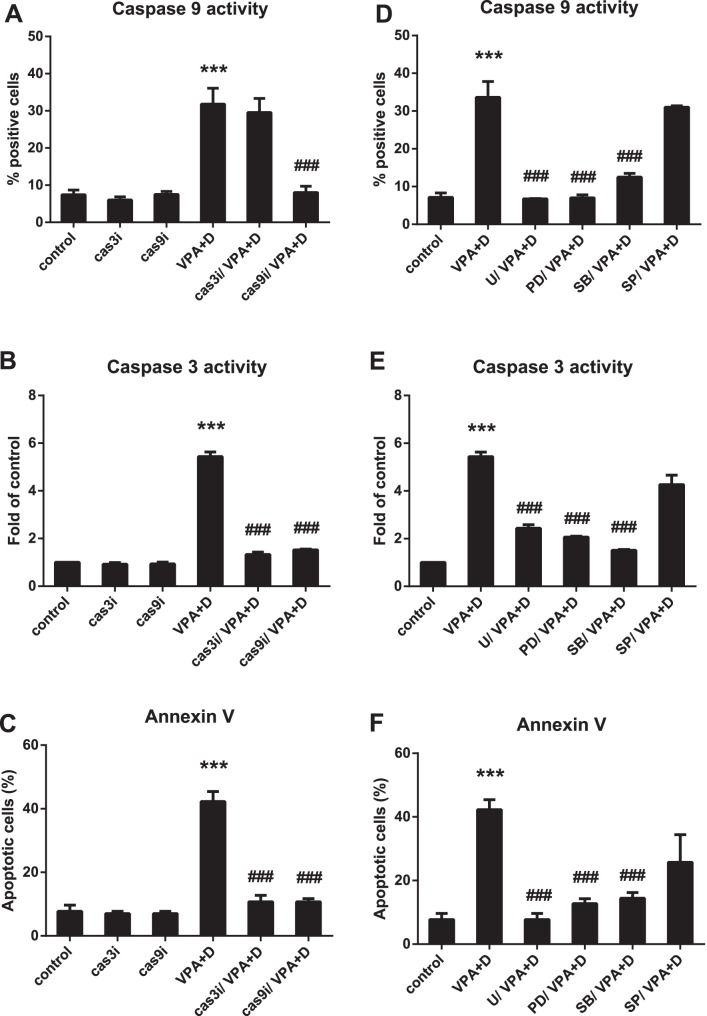
Dasatinib/VPA-induced apoptosis is via a caspase-dependent pathway and depends on MEK/ERK and p38 MAPK. Cells were preincubated with caspase-3 inhibitor (10 µM Z-DEVD-FMK), caspase-9 inhibitor (10 µM LEHD-CHO), MEK/ERK inhibitor (5 µM U0126 and 10 µM PD98059), p38 MAPK inhibitor (10 µM SB203580) and JNK inhibitor (10 µM SP600125) for 1 hr prior to treatment with 0.5 mM of VPA and 5 µM of dasatinib for 72 hr. (A, D) Caspase-9 activity; (B, E) caspase-3 activity (C, F); apoptotic cells. These data represent the means ± SEM. Significantly different from the control (*) or combination of VPA and dasatinib (#); ***, ###: *P*<0.001. Cas3i, caspase-3 inhibitor; cas9i, caspase-9 inhibitor; U, U0126; PD, PD98059; SB, SB203580; SP, SP600125.

Caspase-9, an initiator caspase, forms a complex by binding to apoptotic protease-activating factor-1 (Apaf-1), and then recruits effector caspase-3 [20]. Dasatinib was found to induce the apoptosis of VPA-activated AML cells (Fig. 4) in this research, and thus appears to be associated with caspases. Accordingly, we set out to determine which apoptotic pathway is related to dasatinib/VPA-induced apoptosis. To do so, we pretreated HL60 cells with 10 µM of caspase-3 and -9 inhibitors prior to stimulation with VPA and dasatinib. The activity of each was then measured according to the manufacturer’s protocol, with the combination drug found to markedly increase that of both, as shown in Figures 6A and B. Although the caspase-3 inhibitor did not reduce VPA/dasatinib-induced caspase-9 activity, the caspase-9 inhibitor did reduce combination-induced caspase-3 activity (down to the basal level), thus indicating that caspase-9 is the upstream caspase of caspase-3 (Figs. 6A and B).

Using annexin V staining, we also carried out an experiment to confirm whether caspase-9 and -3 would exert an influence on dasatinib/VPA-induced apoptosis in the same conditions. Both inhibitors were found to block such apoptosis, leading us to conclude that caspase-9 and -3 are essential to the dasatinib/VPA-induced apoptosis pathway in HL60 cells (Fig. 6C). This pathway thus appears to be caspase-dependent (Figs. 6A–C).

### MEK/ERK and P38 MAPK Control Dasatinib/VPA-activated Apoptosis

Two recent studies demonstrated that MAPK is required for dasatinib-elicited AML cell differentiation [21], [22]. To confirm whether MAPK also exerts an effect on dasatinib/VPA-treated HL60 cells, we pretreated these cells with MAPK inhibitors, including 5 µM of U0126, 10 µM of PD98059, 10 µM of SB203580 and 10 µM of SP600125, for 1 h, after which they were stimulated with 0.5 mM of VPA and/or 5 µM of dasatinib. We next measured such dasatinib/VPA-activated apoptotic signals as caspase-9 activity (Fig. 6D), caspase-3 activity (Fig. 6E) and the number of apoptotic cells (Fig. 6F), all three of which were observed to decrease significantly following treatment with MEK/ERK inhibitors U0126 and PD98059 and p38 MAPK inhibitor SB203580. The signals from MEK/ERK and p38 MAPK thus appear to be associated with the initiation of dasatinib/VPA-activated apoptosis (Figs. 6D–F).

## Discussion

AML is characterized by increased leukemic blasts resulting from the deficient development of hematopoietic progenitor and stem cells in bone marrow [23]. The current primary treatment strategy for AML is an intensive course of cytotoxic chemotherapy consisting of induction and consolidation with the aim of achieving and maintaining complete remission (CR) [24], [25]. There is no doubt that postremission therapy is important to helping AML patients to sustain CR [26]. Although CR has been achieved in younger AML patients, they still require hematopoietic cell transplantation as immunotherapy if their risk profile is unfavorable [27]. Timed-sequential induction therapy has been proposed to improve postremission therapy in AML, with all patients achieving remission receiving four cycles of such therapy [28]. Despite these trials and ongoing efforts to improve AML therapy, however, the high post-CR relapse rates and very poor post-relapse survival rates mean a gloomy long-term outlook for this patient group [24]. The development of more effective chemotherapeutic agents is thus a matter of urgency.

Previous studies have shown dasatinib to exert an effect on the differentiation of megakaryocytes [29] and osteoblasts [30]–[32] and the adipogenic differentiation of human multipotent mesenchymal stromal cells [33] and of blasts to neutrophilic granulocytes [34]. It has also been found to induce myeloblast differentiation [22]. Moreover, dasatinib in combination with retinoic acid has been shown to promote AML differentiation [2], [21] and to greatly increase the expression of differentiation marker CD11b. Accordingly, we believe dasatinib has the potential to induce cell differentiation. Recent research has also demonstrated the anti-cancer effects of VPA in several types of cancer cells, although those effects have been found to be more powerful when the drug is combined with such agents as imatinib [14], bortezomib, the first therapeutic proteasome inhibitor [35], selective COX-2 inhibitor celecoxib [36] or radiation [37]. We thus chose VPA to investigate in conjunction with dasatinib in this research. We hypothesized that the differentiation capacity of dasatinib would potentiate VPA-induced apoptosis in AML cell line HL60.

First of all, we investigated the effects of dasatinib and VPA on the cell surface expression of differentiation markers CD11b and CD14 (Fig. 1), with both drugs found to have positive effects on such expression. Surprisingly, following the combined use of the two drugs, the differentiation signal completely disappeared in the AML cells, as shown in Figure 1. At first, the VPA-dasatinib combination seemed to down-regulate the differentiation capacity of each drug. The results presented in Figure 2 revealed 0.5 mM of VPA and 5 µM of dasatinib alone to produce little effect on cell viability in the HL60 cells, whereas their combination significantly inhibited cell proliferation, with cell viability falling below 50% (Fig. 2C). The observed decrease in differentiation markers following the combination treatment may thus have been the result of an increase in apoptosis.

We next searched for the possible mechanism linking apoptosis and differentiation. We stimulated the HL60 cells, with VPA and dasatinib for 48 h, and then monitored them for CD11b or CD14 and annexin V double-positive cells. As shown in Figure S1, the numbers of CD11b/annexin V and CD14/annexin V double-positive cells in the combination group were 1.5- and 1.6-fold higher, respectively, than those in the control group at 48 h, which was in line with our expectations. These cell populations disappeared rapidly thereafter, and we could find no double-positive cells at 72 h. The implication of these findings is that the cell differentiation following combined VPA and dasatinib treatment is the primary contributor to apoptosis initiation, thus confirming our hypothesis that differentiation capacity has an effect on AML cell death. More specifically, the differentiation of CD11b- and CD14-positive cells was accelerated by the combination of the two drugs, which ultimately contributed to apoptosis, thus allowing us to confirm that it was the differentiation capacity of dasatinib-potentiated VPA that induced AML cell apoptosis.

We also observed the VPA-dasatinib combination to exert a strong growth-inhibitory effect on the HL60 cells (Figure 2), and subsequently investigated the possible mechanism of such anti-proliferative activity on cell cycle progression and apoptosis. As shown in Figures 3 and 4, we observed the two drugs to have synergistic effects on both. More specifically, the VPA-dasatinib combination increased the expression of p21^Cip1^ and p27^Kip1^ in the HL60 cells (Fig. 3D), and decreased the expression of G_1_ phase cell cycle regulatory proteins CDK2, 4 and 6 and cyclins D_1_ and E (Figs. 3E and F). Although neither VPA nor dasatinib alone enhanced apoptosis in these cells, their combination produced a powerful apoptotic effect (Figs. 4A and B). We also confirmed the effects of dasatinib and VPA on PBMC and BMC taken from the two patients with AML, and found them to be very similar to those in the HL60 cells (Figs. 4D and E). These results again demonstrate the synergistic effects of the VPA-dasatinib combination on cell viability in AML cells, as shown in Table 1.

Apoptosis, which is considered the ideal form of death for cancer cells, plays an important role in maintaining homeostasis [38]. This type of programmed cell death occurs when the activation of specific pathways results in a series of well-defined morphological events, such as nuclear and cytoplasmic condensation, DNA fragmentation, the exposure of phosphatidylserine residues in the outer plasma membrane leaflet and the release of apoptotic bodies [39], [40]. Dasatinib/VPA-induced apoptosis is also related to nuclear condensation (Fig. 4C). Moreover, apoptotic cell death begins with the release of cytochrome *c* from the mitochondria to form a caspase-activating complex known as the Apaf-1 apoptosome [20]. This complex recruits and activates caspase-9, which then cleaves and activates such downstream caspases as caspase-3 and -7. Caspase-3 cleaves many substrates that respond to DNA strand breaks, such as PARP, eventually leading to apoptosis [41]. We confirmed in this research that the dasatinib-VPA combination evokes apoptosis not only via caspase-9, -3 and -7, but also via the PARP cleavage cascade (Figs. 5 and 6). The powerful combined effects of VPA and dasatinib on apoptosis in AML cells can be seen in the results presented in Table 2.

The most important finding in this research was that the dasatinib/VPA-activated apoptotic signal follows differentiation pathways, such as those of MEK/ERK and p38 MAPK (Figs. 6D and E). Dasatinib alone was found to promote MAPK-dependent cell differentiation and cell cycle arrest in a previous study [21]. We found about 40% of the AML cells in the combination group to have experienced apoptotic death. Differentiation of the cell population through combination treatment may thus hasten the apoptosis of AML cells. Our results also indicate that MEK/ERK and p38 MAPK may be associated with the initiation of such dasatinib/VPA-activated apoptosis (Fig. 6).

We also found the dasatinib-mediated induction of p21^Cip1^ to be blocked by combination treatment with VPA, which is consistent with previous reports [42], [43] indicating that p21^Cip1^ induction decreases following co-treatment with dasatinib and such histone deacetylase inhibitors as sodium butyrate [42] and vorinostat [43]. We also observed the interruption of dasatinib-induced p21^Cip1^ via VPA-potentiated apoptosis, as shown in Figure 4. The inhibitory effect of VPA on dasatinib-induced p21^Cip1^ may contribute to the synergistic apoptotic effects of the combination treatment observed in the HL60 and primary AML cells. It remains unknown whether the inhibitory mechanism of Src and HDAC leads to AML cell death, although there is considerable evidence to suggest that HDAC interference with p21^CIP1^ induction contributes to the potentiation of Src inhibitor-mediated apoptosis, at least in part. In contrast, the loss of p21^CIP1^ has been found to sensitize cells to cytotoxic drugs [44], low doses of cytarabine [45] and various differentiation-inducing agents such as phorbol esters [44]. Given these findings, it is tempting to propose that the interruption of p21^CIP1^ induction in Src inhibitor-treated cells may contribute to enhanced lethality. Direct evidence is lacking at present, however.

We also conducted numerous Western blot experiments on p27^kip^ expression in NB4 and Kasumi-1 cells in an attempt to detect the combined effects of dasatinib and VPA in these cells, but were unable to obtain satisfactory results. Although we observed the poor induction of p27^kip^ in dasatinib/VPA-treated Kasumi-1 cells (data not shown), and found the level of p27^kip^ expression in the Kasumi-1 cells to be lower than that in the NB4 cells, we also observed p27^kip^ expression to have synergistic effects in the Kasumi-1 cells. However, we found measurement of the effect on cell cycle arrest and p27^ kip^ expression in the Kasumi-1 cells to be very difficult, and thus omit the results from the paper, although we considered them to be reasonable. More than 92% of the Kasumi-1 cells and 60% of the NB4 cells experienced apoptotic death following treatment with the dasatinib and VPA combination, as shown in Table 2. Most cells were already dead, and thus it was impossible to detect the p27^ kip^ positive cells or G_1_ phase arrest cells in these samples. That’s why it had a poor induction of p27^ kip^ in combined treatment on NB4 cells, as shown in Figure 3G. In the case of the HL60 cells, in contrast, only 40% died via apoptosis, thus rendering the measurement of cell cycle regulatory proteins such as p27^kip^ easier.

**Figure 7:**
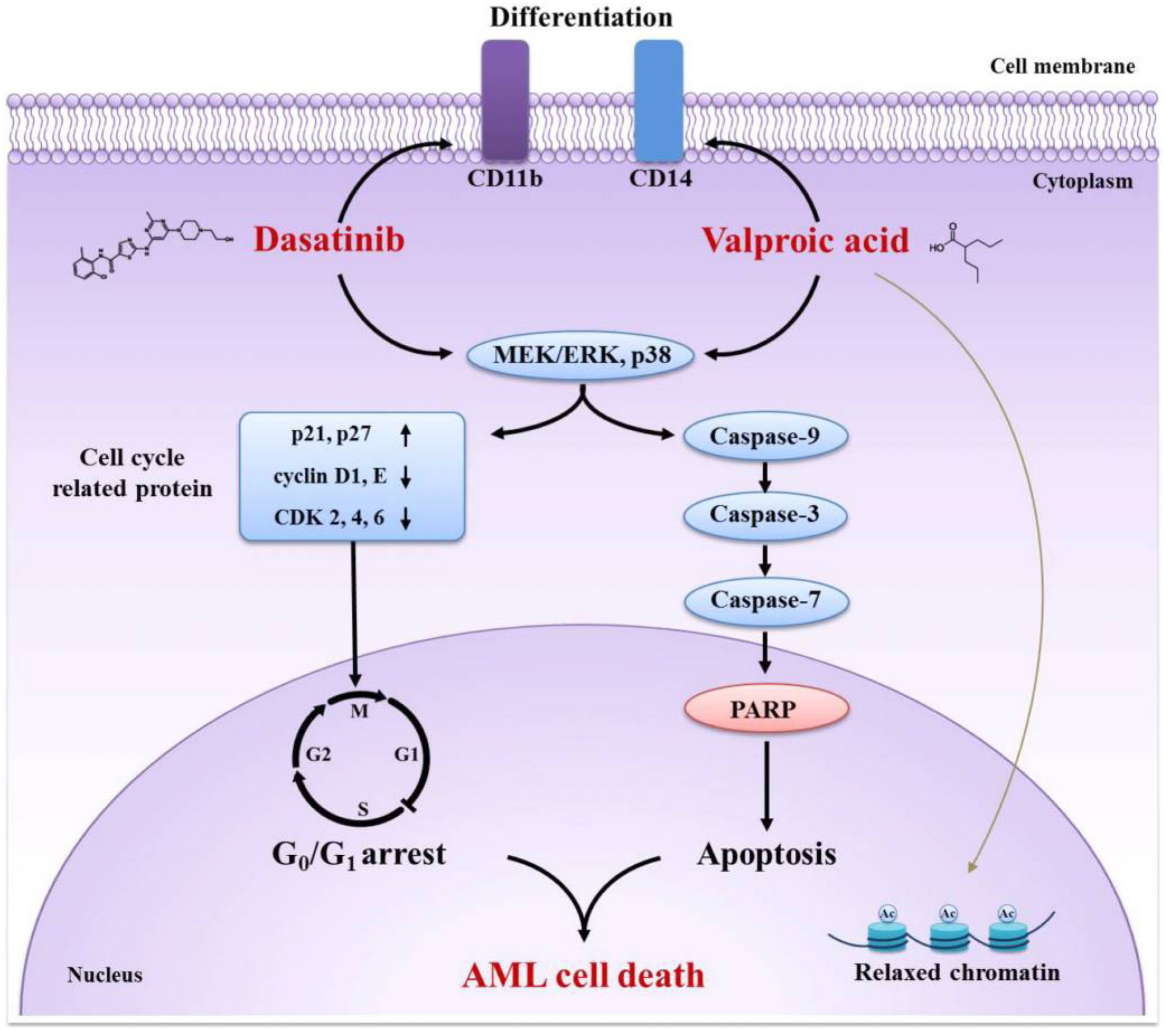
Mechanism by which dasatinib potentiates VPA-treated AML cell death. The combination of dasatinib and VPA on AML cell differentiation capacity is more potent than that of each drug alone. The combination is enough to promote intensive AML cell death through G_1_ cell cycle arrest and caspase-dependent apoptosis. In addition, MEK/ERK and p38 MAPK control dasatinib/VPA-evoked apoptosis as upstream regulators. Eventually, the regulation of cell differentiation capacity contributes to AML cell death.

In conclusion, we found the effect of combined dasatinib-VPA treatment on the apoptotic activity of AML cells to be sufficiently synergistic to promote intensive AML cell death through G_1_ cell cycle arrest and caspase-dependent apoptosis. In addition, our results show MEK/ERK and p38 MAPK to control dasatinib/VPA-induced apoptosis as upstream regulators. Finally, we found that the regulation of cell differentiation capacity contributes to AML cell death. Taken together, our findings indicate that dasatinib accelerates VPA-induced AML cell death through G_1_ arrest and caspase-dependent apoptosis via MEK/ERK and p38 MAPK (Fig. 7). To the best of our knowledge, this is the first study to report that AML cell death is involved in G_1_ arrest and apoptosis following combined treatment with dasatinib and VPA. The results presented herein indicate that combined dasatinib-VPA therapy has a potential role in anti-leukemic treatment.

## Supporting Information

Figure S1 The CD11b^+^/Annexin V^+^ or CD14^+^/Annexin V^+^ cells of combination group were 1.5-fold or 1.6-fold higher than that of control group at 48 hr. The cells were stimulated by VPA and dasatinb for 48 hr, and the CD11b^+^/Annexin V^+^ or CD14^+^/Annexin V^+^ cells were monitored by flow cytometery analysis. These data represent the means ± SEM. Significantly different from control (*) or combination of VPA and dasatinib (#); **, ##: *P*<0.01; ***, ###: *P*<0.001.
(EPS)
